# Semisynthesis of Ribonuclease A using Intein-Mediated Protein Ligation

**DOI:** 10.1100/tsw.2002.855

**Published:** 2002-06-28

**Authors:** Ulrich Arnold, Matthew P. Hinderaker, Ronald T. Raines

**Affiliations:** Department of Biochemistry, University of Wisconsin-Madison, Madison, WI 53706, USA; Department of Chemistry, University of Wisconsin-Madison, Madison, WI 53706, USA; Martin-Luther University Halle-Wittenberg, Department of Biochemistry and Biotechnology, Kurt-Mothes Strasse 3, 06120 Halle, Germany

**Keywords:** intein-mediated protein ligation, semisynthesis, protein chimera, ribonuclease A, peptide synthesis, nonribosomal protein synthesis

## Abstract

The introduction of non-natural amino acid residues or modules into proteins provides a new means to explore the basis for conformational stability, folding/unfolding behavior, or biological function. We exploited intein-mediated protein ligation to produce a semisynthetic ribonuclease A. Of the 124 residues of RNase A, residues 1–94 were linked to an intein. After expression of the fusion protein and thiol-induced cleavage, the RNase A(1–94) fragment possessed a C-terminal thioester. A peptide identical to the C-terminal residues 95–124 of RNase A (with residue 95 being cysteine) was successfully ligated to that thioester thereby reconstituting full-length wild-type RNase A. In mass spectrometry, this semisynthetic RNase A proved to be undistinguishable from the control protein, namely recombinant wild-type RNase A. Recombinant wild-type RNase A was obtained by expression of RNase A(1–124)–intein fusion protein followed by thiol-induced cleavage and hydrolysis of the thioester. Both proteins showed thermal stabilities (T_m_ ) and catalytic activities comparable to the wild-type enzyme, indicating that both proteins folded properly. These results might serve as basis for the semisynthesis of RNase A variants containing non-natural modules in the aforementioned peptide.

## INTRODUCTION

The modification of particular amino acid residues has been used widely as a means to explore the basis for conformational stability, folding/unfolding pathways, and biological function of proteins. While conventional site-directed mutagenesis is restricted to the 20 naturally occurring amino acids, total chemical synthesis is limited to relatively small polypeptides. In contrast, the introduction of non-natural modules into proteins might provide completely new potentialities. Besides site-specific labeling and resistance against proteolysis, these modules could stabilize the protein structure and/or alter folding pathways.

The recently established method of expressed protein ligation (EPL; [1]) or intein-mediated protein ligation (IPL; [2], Fig. 1) extends to whole proteins the older method of native chemical ligation, wherein two chemically synthesized peptides are ligated by use of a C-terminal thioester and an N-terminal cysteine residue[3]. Here, the C-terminal thioester is created by cleavage of a fusion protein consisting of the target protein and an intein in the presence of a thiol reagent. The peptide, which has to start with an N-terminal cysteine residue, is synthesized by standard solid-phase methods allowing the incorporation of a wide variety of nonproteinogenic modules. By this means, studies are not limited by the genetic code and the restrictions due to the ribosomal machinery.

Intein-mediated protein ligation requires a cysteine residue at the ligation site. Ribonuclease A (RNase A; EC3.1.27.5, Fig. 2) has served as model for innumerable studies on protein stability, chemistry, and catalysis[4,5,6,7,8]. Fortunately, the enzyme contains 8 cysteine residues among its 124 residues making it also suitable as a target for studies on the method of intein-mediated protein ligation. Evans et al.[2] used cysteine residue 110 to reconstitute wild-type RNase A. Hondal et al.[9] also used Cys110 for ligation experiments but incorporated L-selenocysteine instead of cysteine. We used Cys95 for the experiments described below and, hence, a 30mer peptide, which would allow the incorporation of non-natural modules at more sites.

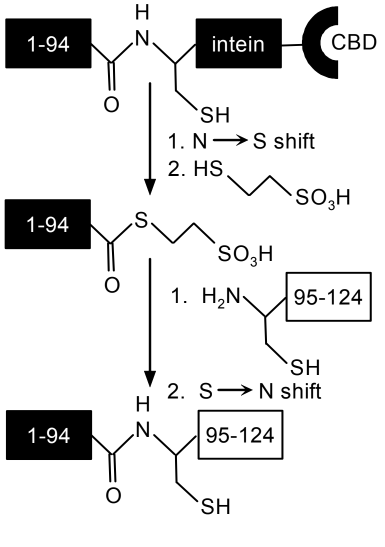

**FIGURE 1.** Scheme for semisynthesis of RNase A by intein-mediated protein ligation. N-terminal part (1–94, black box) is produced by biosynthesis, C-terminal part (95–124, white box including Cys95) is produced by chemical synthesis.

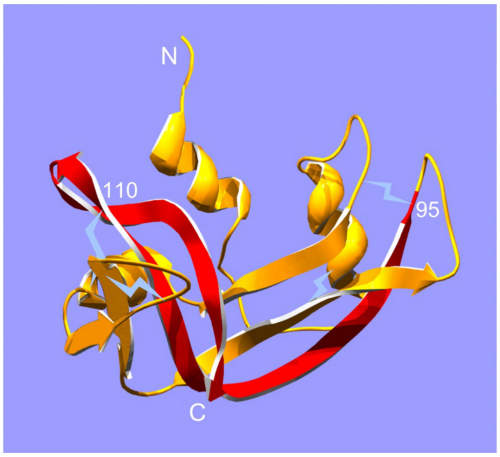

**FIGURE 2.** RNase A fragment 1–94 (orange, with an additional Met-1, not shown) is produced by biosynthesis. In intein-mediated protein ligation, it is ligated to RNase A fragment 95–124 (red), which is produced by chemical synthesis and can contain non-natural modules.

## RESULTS AND DISCUSSION

DNA sequences encoding both full-length RNase A (from plasmid pBXR[10]) and its 1–94 fragment were inserted into plasmid pTXB1 (New England Biolabs) by standard techniques using the PCR. Cultures of *E. coli* ER2566 (New England Biolabs) containing the plasmids were grown as described by the manufacturer. It is noteworthy that both expressed proteins produced by this method (fused to the *mxe*-intein–chitin binding domain) possess an additional methionine residue at their N-terminus: Met-1.

Expression of the fusion proteins yielded about 25 mg/l of bacterial culture corresponding to a theoretical amount (with 100% intein cleavage) of about 8 mg of RNase A(1–124) or RNase A(1–94) per liter of bacterial culture. Growth of the cultures at 25°C resulted in soluble fusion protein that effectively bound to the chitin resin (Fig. 3).

After production of the thioester-tagged proteins (with 2-mercaptoethanesulfonic acid), “recombinant” RNase A(1–124) was subjected to folding directly[10] whereas the RNase A(1–94)~thioester was precipitated by sodium deoxycholate/trichloroacetic acid[11]. The precipitate was dissolved in 2 M guanidine–HCl and the peptide, which had been synthesized by solid-phase

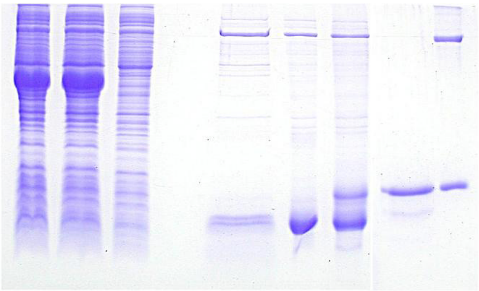

**FIGURE 3.** SDS-PAGE gel (15% w/v) of samples from the production of semisynthetic RNase A: 1, crude cell extract; 2, soluble fraction; 3, chitin column flow through; 4, chitin column wash, 5, eluted RNase A(1–94); 6, concentrated sample #5; 7, ligation reaction; 8, purified ligation product, namely semisynthetic RNase A; 9, molecular weight marker: BSA (67 kDa), RNase A (13.8 kDa).

**TABLE 1**
**Properties of RNase A Produced by Three Distinct Methods**

| RNase A Origin | Molecular Mass (Da) | | | $T_m$ (°C)[1] | $k_{cat}/K_m$ ($10^7$ $M^{-1}$ $s^{-1}$)[2] |
|---|---|---|---|---|---|
| | Expected | MALDI-MS | ESI-MS | | |
| Bovine pancreas[3] | 13,683 | ND | ND | 63.6 ± 0.2 | 1.5 ± 0.2 |
| Recombinant | 13,813 | 13,815 | 13,818 | 63.5 ± 0.1 | 1.4 ± 0.3 |
| Semisynthetic | 13,813 | 13,809 | 13,813 | 63.4 ± 0.2 | 1.5 ± 0.2 |

[1] Values were determined by UV spectroscopy in 50 mM sodium phosphate buffer (pH 8.0) containing NaCl (25 mM).
[2] Values were determined at 25°C with 6-carboxyfluorescein~dArU(dA)2~6-TAMRA as substrate[12].
[3] Purchased from Sigma Chemical.

synthesis and contained the sequence CAYKTTQANKHIIVACEGNPYVPVHFDASV, was added at a 40-fold molar excess. Ligation was allowed to proceed for at least 16 h at pH 8 and room temperature. The ligation yield, however, was rather low (about 1% with respect to the molar quantity of peptide). Afterwards, the ligation reaction was diluted to ≤ 0.1 mg protein per milliliter and the ligation product, named "semisynthetic RNase A", was subjected to folding[10]. Folded proteins were purified by affinity chromatography on GDP~Sepharose and cation exchange chromatography on sulfopropyl Sepharose resin (Fig. 3).

After purification, both proteins were characterized by mass spectrometry (Table 1). The results show that both proteins are undistinguishable from one another. However, both proteins possess an additional Met-1 in comparison to wild-type RNase A from bovine pancreas. The transition temperature ($T_m$) indicates the conformational stability of a protein. The comparison of the $T_m$ of a mutant enzyme or enzyme variant with that of the wild-type enzyme indicates whether the manipulations affect the conformational stability of the protein molecule. On the other hand, enzymatic activity provides an extremely sensitive measure of the formation of the native protein structure. The determination of $T_m$ and the catalytic parameters ($k_{cat}/K_m$) proved that both protein proteins folded properly and show characteristics comparable to those of wild-type RNase A from bovine pancreas (Table 1).

Intein-mediated protein ligation enables the semisynthetic incorporation of a wide variety of nonproteinogenic modules. Thus, based on the results presented here it is feasible to exploit the whole palette of chemical synthesis to modify the C-terminal part of RNase A as well as parts of other proteins.

## ACKNOWLEDGEMENT

We are grateful to Dr. R.J. Hondal for help and advice. U.A. was supported by the Arthur B. Michael postdoctoral fellowship. M.P.H. was supported by NIH Chemistry-Biology Interface Training Grant GM08506. This work was supported by NIH Grant GM44783 to R.T.R.

## References

1. Muir, T.W., Sondhi, D., and Cole, P.A. (1998) Expressed protein ligation: a general method for protein engineering. *Proc. Natl. Acad. Sci. U. S. A.* **95**, 6705–6710.
2. Evans, T.C., Jr., Benner, J., and Xu, M.Q. (1998) Semisynthesis of cytotoxic proteins using a modified protein splicing element. *Protein Sci.* **7**, 2256–2264.

3. Dawson, P.E., Muir, T.W., Clark-Lewis, I., and Kent, S.B. (1994) Synthesis of proteins by native chemical ligation. *Science* **266**, 776–779.
4. Pace, C.N., Laurents, D.V., and Thomson, J.A. (1990) pH dependence of the urea and guanidine hydrochloride denaturation of ribonuclease A and ribonuclease T1. *Biochemistry* **29**, 2564–2572.
5. Houry, W.A., Rothwarf, D.M., and Scheraga, H.A. (1994) A very fast phase in the refolding of disulfide-intact ribonuclease A: implications for the refolding and unfolding pathways. *Biochemistry* **33**, 2516–2530.
6. Arnold, U., Rücknagel, K.P., Schierhorn, A., and Ulbrich-Hofmann, R. (1996) Thermal unfolding and proteolytic susceptibility of ribonuclease A. *Eur. J. Biochem.* **237**, 862–869.
7. Arnold, U. and Ulbrich-Hofmann, R. (2000) Differences in the denaturation behavior of ribonuclease A induced by temperature and guanidine hydrochloride. *J. Protein Chem.* **19**, 345–352.
8. Raines, R.T. (1998) Ribonuclease A. *Chem. Rev.* **98**, 1045–1066.
9. Hondal, R.J., Nilsson, B.L., and Raines, R.T. (2001) Selenocysteine in native chemical ligation and expressed protein ligation. *J. Am. Chem. Soc.* **123**, 5140–1541.
10. delCardayré, S.B., Ribó, M., Yokel, E.M., Quirk, D.J., Rutter, W.J., and Raines, R.T. (1995) Engineering ribonuclease A: production, purification and characterization of wild-type enzyme and mutants at Gln11. *Protein Eng.* **8**, 261–273.
11. Arnold, U. and Ulbrich-Hofmann, R. (1999) Quantitative protein precipitation from guanidine hydrochloride-containing solutions by sodium deoxycholate/trichloroacetic acid. *Anal. Biochem.* **271**, 197–199.
12. Kelemen, B.R., Klink, T.A., Behlke, M.A., Eubanks, S.R., Leland, P.A., and Raines, R.T. (1999) Hypersensitive substrate for ribonucleases. *Nucleic Acids Res.* **27**, 3696–3701.
